# Oxygen Kinetics and Heart Rate Response during Early Recovery from Exercise in Patients with Heart Failure

**DOI:** 10.1155/2012/512857

**Published:** 2012-01-24

**Authors:** Charalampos D. Kriatselis, Sotirios Nedios, Sebastian Kelle, Sebastian Helbig, Martin Gottwik, Christian von Bary

**Affiliations:** ^1^The Department of Internal Medicine Cardiology, Deutsches Herzzentrum Berlin, Augustenburger Platz 1, 13353 Berlin, Germany; ^2^Department of Internal Medicine-Cardiology, Klinikum Nürnberg, Nuremberg, Germany; ^3^Klinik und Poliklinik für Innere Medizin II, University Medical Center Regensburg, Regensburg, Germany

## Abstract

*Background*. The purpose of this study was to assess the post-exercise O_2_ uptake and heart rate response in patients with heart failure (HF) in comparison to healthy individuals. *Methods and Results*. Exercise testing of all subjects was conducted according to the RITE-protocol. The study subjects were classified according to their peak oxygen uptake (peak VO_2_) in four groups: healthy individuals with a peak VO_2_ >22 mL/kg/min (group 1, *n*: 50), and patients with HF and a peak VO_2_ of 18–22 mL/kg/min, (group 2, *n*: 48), 14–18 mL/kg/min (group 3, *n*: 57), and <14 mL/kg/min (group 4, *n*: 31). Both peak VO_2_ and HR declined more slowly in the patients with HF than in the normal subjects. Recovery of VO_2_ and HR followed monoexponential kinetics in the early post-recovery phase. This enabled the determination of a time constant for both HR and VO_2_ (TC VO_2_ and TC HR). From group 1 to 4 there was a prolongation of the time constant for VO_2_ and HR: TC VO_2_ (group 1: 110 ± 34, group 2: 197 ± 43, group 3: 238 ± 80, and group 4: 278 ± 50 sec), and TC HR (group 1: 148 ± 82, group 2: 290 ± 65, group 3: 320 ± 58, and group 4: 376 ± 55 sec). *Conclusion*. The rate of decline of VO_2_ and HR in the early post-exercise phase is inversely related to the peak VO_2_. The time constant for oxygen uptake (TC VO_2_) and heart rate (TC HR) might prove a useful parameter for more precise monitoring and grading of HF.

## 1. Introduction

Oxygen uptake kinetics and heart rate response during exercise have been extensively studied in both normal subjects and patients with heart failure [1–3]. Anaerobic threshold and maximal oxygen uptake are reliable parameters for assessing the functional capacity of an individual and determining the progression of heart failure, response to medication, and overall prognosis [4–6]. Exercise and the early postexercise phase can be considered as a continuum, and symptoms such as dyspnea and fatigue appearing during exercise are prominent also in the first post-exercise minutes. Cole et al. [7] showed that a delayed decrease in the heart rate during the first minute after graded exercise is a powerful predictor of overall mortality, independent of changes in heart rate during exercise. Similar results were published by Tang et al. [8] in heart failure patients and by Lacasse et al. [9] in patients with obstructive lung disease. De Groote et al. [10] found that kinetics of oxygen consumption during recovery was delayed in patients with dilated cardiomyopathy and that the ratio between total oxygen consumption during exercise and recovery was an independent prognostic marker in patients with moderate exercise intolerance. The purpose of this study was to compare heart rate and oxygen consumption in the early recovery phase after maximal, symptom-limited exercise in patients with different functional stages of heart failure and in healthy individuals.

## 2. Methods

### 2.1. Study Population

The study included 136 patients with heart failure referred for symptom-limited cardiopulmonary exercise testing as well as 50 age-matched healthy individuals. The diagnosis of heart failure was made on the basis of history taking, clinical evaluation and estimation of systolic pump function of the left ventricle (ejection fraction <40% measured by echocardiography and/or left ventriculography). Patients were excluded if they could not exercise maximally for noncardiac reasons such as severe pulmonary disease, peripheral vascular occlusive disease and orthopedic or neurological disease. Patients with a pacemaker were also excluded. Patients' medication was not withdrawn before the test, and no patient was taking part in a regular exercise program. The study conforms with the principles outlined in the Declaration of Helsinki. The baseline demographic and clinical characteristics are shown in Table 1.

### 2.2. Cardiopulmonary Exercise Testing

Exercise testing of all patients was conducted according to the Ramping Incremental Treadmill Exercise (RITE) Protocol [11]. In this symptom-limited treadmill exercise protocol initial elevation and velocity are 4% and 1 mph, respectively. After each minute of exercise, elevation increases by 1% and velocity by 0.5 mph. Patients were encouraged to exercise until symptoms were intolerable. Investigator-determined exercise end points were nonsustained ventricular tachycardia (>5 beats), any sustained arrhythmia, high-degree AV block, ST-segment depression >3 mm, systolic blood pressure >230 mmHg, or progressive decrease in blood pressure.

Exhaled gases were analyzed on a breath-by-breath basis using a respiratory gas analyzer (MedGraphics Cardio O_2_, CA, USA). Oxygen uptake (VO_2_), carbon dioxide output (VCO_2_), tidal volume (VT), and breathing rate were measured.

### 2.3. Cardiopulmonary Parameters

From the above data, minute ventilation (VE), respiratory exchange ratio (VCO_2_/VO_2_), the O_2_ pulse (VO_2_/HR), and the ventilatory equivalents for O_2_ and CO_2_ (VE/VO_2_, VE/VCO_2_) were calculated. Peak VO2 was determined as the highest VO_2_ achieved during exercise.

The anaerobic threshold was measured with the V-slope method [12]. Typical changes in ventilatory equivalents and end-tidal gas concentrations (PET O_2_ and PET CO_2_) were examined to search for agreement in cases that were questionable with regard to the precise anaerobic threshold values. Predicted maximal VO_2_ was determined by using the regression equations of Wasserman et al. [13]. Functional capacity (FC) was defined as the percentage of the predicted (for sex, age, and body weight) maximal VO_2_ reached. Post-exercise heart rate and oxygen consumption kinetics were described using a time constant (TC).

**Figure 1:**
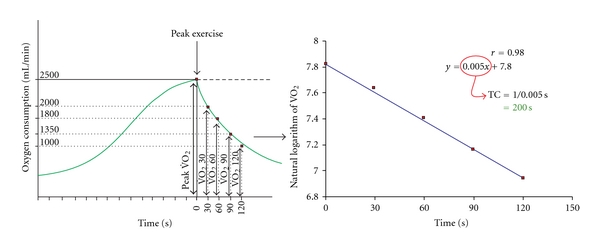
Example of calculation of the time constant (TC) value for the first 2 postexercise minutes. TC expresses the time (in sec) after which VO_2_ has declined 63% below the peak value.

To quantify the time course of the VO_2_ decrease, we calculated the natural logarithm of VO_2_ at 5 points: peak VO_2_ and VO_2_ at 30, 60, 90, and 120 sec of the post-exercise period. The linear regression of the natural logarithm over time was expressed as *y* = − *A* · *x* + *B*, (*A*, *B* = constant). The time constant was then obtained by −1/*A* and was expressed as TC VO_2_ (s) (Figure 1). In a similar manner, the time constant for heart rate decrease TC HR was calculated. The correlation constant *r* showed the level of correlation of post-exercise VO_2_ or HR with a monoexponential curve.

The study subjects were classified according to their peak VO_2_ into four groups: group 1 with a peak VO_2_ > 22 mL/kg/min (*n*: 50), group 2 with 18–22 mL/kg/min, (*n*: 48), group 3 with 14–18 mL/kg/min (*n*: 57), and group IV with <14 mL/kg/min (*n*: 31), respectively.

### 2.4. Statistical Analysis

Continuous variables are expressed as mean value ± standard deviation and are compared using an unpaired *t*-test. A *P* value of <0.05 was required for statistical significance.

## 3. Results

The four study groups were similar with respect to age and sex distribution. A total of 136 patients and all 50 healthy subjects exercised until the peak exercise level, defined as the exercise level at which the subject reached at least 80% of predicted maximal heart rate and 50% of his or her respiratory reserve. The clinical characteristics of the patients are shown in Table 1.

For TC VO_2_, the correlation coefficient with a monoexponential curve was above 0.97 (mean ± SD = 0.98 ± 0.01) in the 50 normal controls and above 0.95 (0.97 ± 0.02) in the 136 patients. Correlation coefficient for TC HR was above 0.95 (0.98 ± 0.02) in controls and above 0.95 (0.97 ± 0.03) in patients. We found no patient with a correlation coefficient of less than 0.90 for either TC VO_2_ or TC HR. High values of the correlation coefficient indicate that both VO_2_ and HR declined monoexponentially in the first 2 minutes of recovery. After the first 2 minutes, neither heart rate nor oxygen consumption curves were monoexponential and for that reason no comparison between the different groups could be made for this period of time.

Peak heart rate differed significantly between the four groups and was lower in the groups with reduced functional capacity (Table 2). The rate of decline of heart rate and oxygen consumption was inversely related to the functional capacity. In normal subjects, heart rate and oxygen consumption declined rapidly as this was expressed by a low TC (TC-VO_2_  110 ± 34, TC-HR 148 ± 82 sec), and 90% of them had reached at least 1/2 of the base-line heart rate and oxygen consumption by 120 sec in the recovery period. In the patients with heart failure, heart rate and oxygen consumption declined more slowly than in normal subjects. The rate of decline was slower with a reduced functional capacity. By 120 sec in the recovery period, 90% of the patients in group 2 had reached at least 1/2 of the base-line heart rate and oxygen consumption, while this was the case in only 50% and 20% in groups 3 and 4, respectively. Patients on beta-blockers tended to have a lower peak heart rate than patients without beta-blocking medication: peak HR in bpm, FC 70–90:  128 ± 10  versus 134 ± 12, *P*:  0.08, FC 50–70:  118 ± 9  versus 126 ± 12, *P*: 0.07, and FC <50:  106 ± 9  versus 111 ± 12, *P*: 0.09. There was no difference in the time constants for heart rate and oxygen consumption: TC for HR in sec: FC 70–90:  285 ± 66  versus 291 ± 64, *P*: 0.56, FC 50–70:  323 ± 62  versus 318 ± 50, *P*: 0.33, FC <50:  374 ± 58  versus 377 ± 52, *P*: 0.23. TC for VO_2_ in sec: FC 70–90 : 199 ± 45 versus 198 ± 43, *P*: 0.25, FC 50–70:  240 ± 80  versus 237 ± 80, *P*: 0.43, FC <50:  280 ± 52 versus 277 ± 50, *P*: 0.44.

## 4. Discussion

Patients with clinically manifest heart failure have a decreased exercise capacity. During exercise, symptoms such as dyspnea and fatigue appear in relatively early stages and account for termination of exercise before maximum levels are reached. The severity of the impairment has been quantified by several types of exercise testing. Among the parameters, anaerobic threshold, peak VO_2_, and the ratio of the increase in VO_2_ to the increase in work rate (ΔVO_2_/ΔWR), which are obtained from respiratory gas exchange analysis, have been employed by many investigators because of their good sensitivity and noninvasive mode of determination.

In this study, we found that patients with heart failure compared to normal individuals show a different response of heart rate and oxygen uptake not only during exercise but also in the first minutes of recovery. For the first 2 minutes after the cessation of exercise, the VO_2_ time relationship declined monoexponentially. During this early period of recovery oxygen uptake declines rapidly and the rate of decline seems to be related to the resynthesis of phosphocreatine and reloading of myoglobin and hemoglobin with oxygen [14]. Thus, one of the reasons for the slower decline of recovery, oxygen uptake in patients with heart failure seems to be the impaired ability of the circulatory system to deliver rapidly the amount of oxygen needed for replenishment of myoglobin in the peripheral muscles. This is supported by the study of Tanabe et al. [15] in which slower recovery kinetics of VO_2_ and VCO_2_ correlated very well with the impairment of circulatory response to exercise and delayed recovery of cardiac output. The fact that peak VO_2_ and indexes of recovery did not correlate with ejection fraction of left ventricle at rest was also shown by Tang et al. [8] and emphasizes that a dynamic interplay of cardiac muscle contractility, heart rate, peripheral vascular resistance, and venous filling takes place during exercise and the recovery phase, rendering left ventricular ejection fraction at rest a poor predictor of exercise capacity. Another factor that possibly contributes to the slower recovery kinetics of VO_2_ in heart failure is the impaired oxygen transport across the capillary-alveolar membrane in the lungs. It is known that significant alterations of pulmonary function occur in heart failure patients [16]. These include increased pulmonary restriction, ventilation-perfusion mismatch with an increase of the physiological dead space, and increased respiratory effort for a given workload compared to a healthy population. Such alterations are believed to result from fluid accumulation in the interstitial space secondary to increased pulmonary capillary pressure and are more pronounced during exercise. Elevation of VE/VCO_2_, which reflects an increase in ventilatory dead space, was found to correlate with a decreasing functional capacity in this and other studies [17, 18]. Because VO_2_ is related to oxygen extraction as well as cardiorespiratory function, the prolongation of VO_2_ decline during recovery may result also from the altered utilization of oxygen in muscles and other organs. It is known that patients with heart failure have reduced oxygen reserves, impaired oxygen transport, and muscle metabolism as well as muscle atrophy [19–21]. Cohen-Solal et al. [22] found a good correlation between the recovery half-time of oxygen consumption and the recovery half-time of the Pi/PCr ratio of the anterior leg muscle in patients with heart failure.

In this study, heart rate at rest and at maximal exercise was significantly lower in healthy subjects than in patients with heart failure. The differences in resting heart rate may well be attributed to the decrease of vagal tone in combination with an increased adrenergic tone, which occur as some of the earlier neural compensatory mechanisms in the course of heart failure. At comparable exercise stages and in the absence of chronotropic incompetence, heart rate in heart failure patients tends to be higher than in healthy subjects. Heart rate “overshoot” is a compensatory mechanism for the impaired augmentation of stroke volume of the failing myocardium during increasing exercise. Patients with heart failure reach their maximal exercise capacity in earlier exercise stages, than age- and sex-matched healthy subjects. This is one of the reasons to explain the lower maximal heart rate in heart failure patients observed in this and other [8] studies. Patients with *β*-blocking medication tend to have lower maximal heart rates than patients without. However, in this study the difference did not reach statistical significance, probably because of the relatively low dose of ß-blockers used (about 1/4 to 1/8 of the currently maximal recommended dose).

In this study, recovery of heart rate in patients with mild heart failure (functional capacity 70–90%) lasted approximately twice as long as in healthy individuals (TC HR: 290 ± 65 versus 148 ± 82 sec). Further prolongation of heart-rate recovery kinetics was observed with decreasing functional capacity. Imai et al. [23] examined the physiologic characteristics of heart rate recovery after exercise in healthy adults, athletes, and patients with heart failure. They demonstrated that in all three groups, vagal reactivation was the principal determinant of the decrease of heart rate during early recovery. This mechanism is independent of age and intensity of exercise. In heart failure-impaired vagal reactivation causes a slower decrease of heart rate during recovery. Considering the relationship between autonomic tone imbalance and cardiac mortality [24, 25], it is not surprising that a delayed decrease of heart rate during recovery from exercise is a powerful predictor of mortality [7].

The role of sympathetic nervous system in heart failure is pivotal. In their interesting work, Lambert et al. [26] examined the cardiac and whole body sympathetic nervous system activity in patients with severe heart failure and have been able to demonstrate an association between the increased release of central nervous system monoamine neurotransmitters and the sympathoexcitation that is variably present in treated heart failure patients. In their work, Kaye et al. [27] quantified whole-body and cardiac sympathetic activity in patients with heart failure and correlated them to clinical outcome, thus providing evidence for the deleterious effect of sympathetic activation in heart failure. Finally, Hasking et al. [28] showed that in patients with heart failure continuously increased sympathetic tone can induce an end-organ refractoriness to adrenergic stimulation, as expressed by a blunted response to exercise. All these studies underpin the use of beta-blockers in heart failure [29].

**Figure 2:**
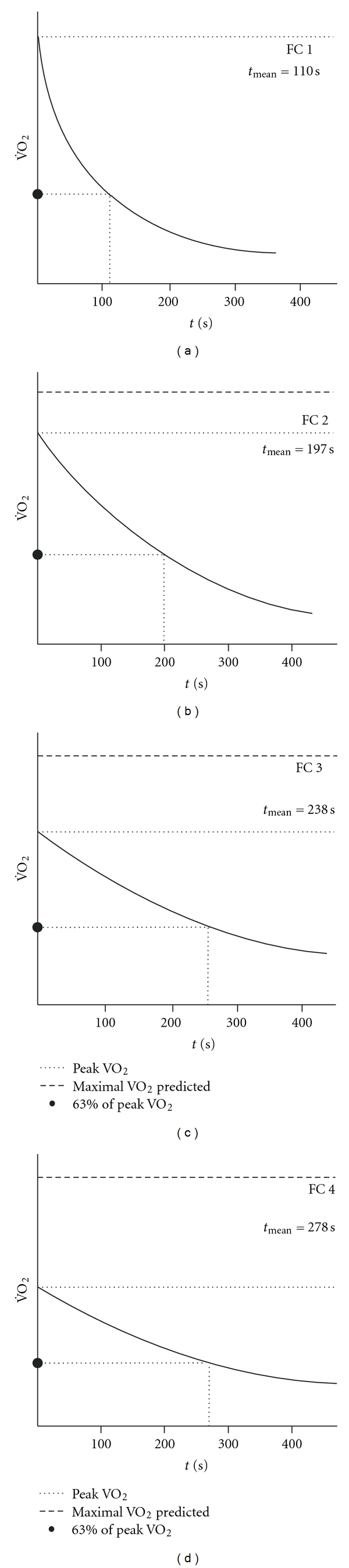
Schematic presentation of the mean VO_2_ recovery curves for the four groups.

**Figure 3:**
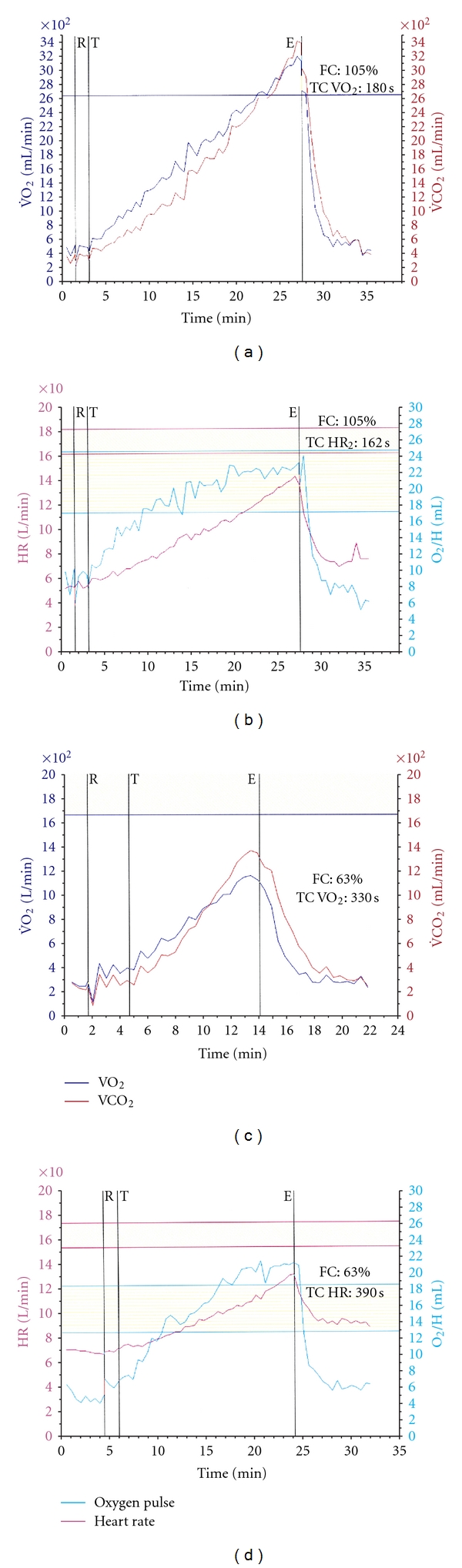
Examples of VO_2_ and HR recovery after exercise with the corresponding time constant in a normal individual (a), (b) and in a patient with heart failure (c), (d).

 In our study, patients with heart failure that were on beta-blockers had tendentially lower peak heart rates than patients without beta blocking medication. Beta-blockade did not influence the time constants for heart rate and VO_2_ recovery among patients of the same functional class (Figure 2). This finding is in accordance with other studies that have shown also a negligible effect of beta-blockade on heart rate recovery after exercise (Figure 3) [30, 31]. This supports the hypothesis that although heart failure patients have a high-resting sympathetic tone as reflected by the elevated resting heart rates, decrease of heart rate after maximal exercise is influenced primarily by the activation of vagal tone, while prolonged sympathetic activation seems to play a minor role at least during early recovery period.

## 5. Limitations

We assessed the kinetics of oxygen uptake and heart rate during early recovery after symptom-limited maximal exercise by the use of a ramping incremental protocol. How TC VO_2_ and TC HR change during other types of exercise, especially in submaximal exercise or by using a bicycle ergometer, remains to be determined.

The number of patients with heart failure who were under *β*-blocking medication was relatively small, and the dose used was lower than currently recommended. For that reason, the effect of ß-blockade on VO_2_ kinetics and heart rate response cannot be evaluated.

## 6. Conclusions

Reduction of functional capacity is associated with prolongation of the early recovery of oxygen consumption and heart rate after maximal exercise. The time constants TC VO_2_ and TC HR characterize the monoexponential decline of oxygen consumption and heart rate in the first 2 minutes of recovery and might be useful parameters in the diagnosis and management of heart failure.

##  Conflict of Interests

The authors declare that they have no conflict of interests.

## Figures and Tables

**Table 1:** Clinical characteristics of 136 patients who achieved peak exercise level.

Age (yr)				51 ± 13
Range				24–70
Men/women				89/61
Etiology of heart failure				
CAD				92 (67%)
Idiopathic dilated cardiomyopathy				53 (39%)
Valvular disease				5 (4%)
Left ventricular ejection fraction (%)				30 ± 12
NYHA functional class (FC)				
II				48 (35%)
III				57 (42%)
IV				31 (23%)

Medication (%)				
	ACE inhibitors	Beta-blockers	Diuretics	
FC II	75	30	89	
FC III	88	28	97	
FC IV	86	17	98	

Data are expressed as mean value ± SD or number of patients, unless otherwise indicated.

**Table 2:** Results of exercise testing.

(%)	*n*	AT (mL/min/kg)	Peak VO_2_ (mL/min/kg)	ΔVO_2_/ΔWRs (mL/min/W)
FC >90	50	18.4 ± 6	31 ±7	11.6 ± 0.9
FC 70–90	48	14 ± 4	22 ± 4	9.6 ± 0.8
FC 50–70	57	8 ± 3	17 ± 4	8.5 ±1.0
FC <50	31	6 ± 2	10 ± 2	5.2 ± 1.3

(%)	VE/VCO_2_ Peak ex.	Peak HR (bpm)	TC (VO_2_) (sec)	TC (HR) (sec)

FC >90	27 ± 2.8	154 ± 22	110 ± 34	148 ± 82
FC 70–90	31 ± 3.5	132 ± 11	197 ± 43*	290 ± 65*
FC 50–70	37 ± 5.6	123 ± 12	238 ± 80*	320 ± 58*
FC <50	45 ± 7.2	110 ± 10	278 ± 50*	376 ± 55*

*n* : number of cases, AT: anaerobic threshold, peak VO_2_: oxygen uptake at peak exercise, ΔVO_2_/ΔWR: ratio of the increase in oxygen uptake to the increase in work rate, VE/VCO_2_ peak Ex.: respiratory equivalent for carbon dioxide at peak exercise, peak HR: heart rate at peak exercise, TC (VO_2_) and TC (HR): time constant for the postexercise oxygen uptake and heart rate, respectively. *P* < 0.05 for all parameters of each group compared to the previous group (i.e., FC <50% versus FC 50–70%, FC 50–70% versus FC 70–90%, and FC 70–90% versus FC >90%). Values are expressed as means ± SD. *P < 0.05 for the comparison with the previous groups.
